# Sex reversal assessments reveal different vulnerability to endocrine disruption between deeply diverged anuran lineages

**DOI:** 10.1038/srep23825

**Published:** 2016-03-31

**Authors:** Stephanie Tamschick, Beata Rozenblut-Kościsty, Maria Ogielska, Andreas Lehmann, Petros Lymberakis, Frauke Hoffmann, Ilka Lutz, Werner Kloas, Matthias Stöck

**Affiliations:** 1 Leibniz-Institute of Freshwater Ecology and Inland Fisheries (IGB), Müggelseedamm 301 & 310, D-12587 Berlin, Germany; 2 Department of Evolutionary Biology and Conservation of Vertebrates, Wroclaw University, Sienkiewicza 21, 50-335 Wroclaw, Poland; 3 Federal Institute for Materials Research and Testing (BAM), Richard-Willstätter-Str. 11, D-12489 Berlin, Germany; 4 Natural History Museum of Crete, University of Crete, Knossou Ave., 71409 Heraklion, Crete, Greece

## Abstract

Multiple anthropogenic stressors cause worldwide amphibian declines. Among several poorly investigated causes is global pollution of aquatic ecosystems with endocrine disrupting compounds (EDCs). These substances interfere with the endocrine system and can affect the sexual development of vertebrates including amphibians. We test the susceptibility to an environmentally relevant contraceptive, the artificial estrogen 17α-ethinylestradiol (EE2), simultaneously in three deeply divergent systematic anuran families, a model-species, *Xenopus laevis* (Pipidae), and two non-models, *Hyla arborea* (Hylidae) and *Bufo viridis* (Bufonidae). Our new approach combines synchronized tadpole exposure to three EE2-concentrations (50, 500, 5,000 ng/L) in a flow-through-system and pioneers genetic and histological sexing of metamorphs in non-model anurans for EDC-studies. This novel methodology reveals striking quantitative differences in genetic-male-to-phenotypic-female sex reversal in non-model vs. model species. Our findings qualify molecular sexing in EDC-analyses as requirement to identify sex reversals and state-of-the-art approaches as mandatory to detect species-specific vulnerabilities to EDCs in amphibians.

Amphibians face a global ongoing decline^1^,^2^. Anthropogenic causes such as industrial agriculture^3^, habitat destruction^4^,^5^, invasive species^6^, climate change^7^, land use^8^ and infectious diseases^9^, including several forms of chytridiomycosis^10^,^11^, are among the major threats. However, the sum of multiple stressors^1^,^7^, some of which poorly known, is considered to be the true reason for the massive population declines. One potential cause represents endocrine disrupting compounds (EDCs)^12^. Besides pesticides, EDCs comprise either natural products or synthetic chemicals that mimic, enhance (an agonist), or inhibit (an antagonist) the action of hormones and in this way interfere with the synthesis, secretion, transport, binding, action, or elimination of natural hormones, which are responsible for the maintenance of homeostasis, reproduction, development, and/or behavior^13^. Considerable amounts of EDCs are globally found in waste and surface waters^14^,^15^ and can easily enter the body of aquatic organisms and impair their natural hormonal pathways. EDCs are well known for their negative impacts on the sexual development of aquatic organisms such as fish^16^,^17^ and are suspected to cause fertility problems in humans^18^,^19^. However, their impact to non-model amphibians with aquatic larvae is not well studied, despite recent evidence for high EDC-relevance to suburban frog populations^20^. One globally relevant EDC is 17α-ethinylestradiol (EE2), a synthetically stabilized estrogen and main ingredient of many female contraceptive pills. The inert EE2 is then excreted and insufficiently eliminated by sewage plants and hence reaches aquatic ecosystems^14^. It is a main hormonal pollutant, resistant to degradation, that accumulates in sediments and biota^14^. Concentrations from 24 to 831 ng/L have been detected in European and American surface waters^21^,^22^,^23^. Such concentrations have been shown to alter behavior and somatic and sexual development in fish and amphibians^12^,^14^,^15^. Due to their semi-aquatic life cycle, often aquatic reproduction and a highly permeable skin, amphibians are especially sensitive to EDCs. Effects on development and reproduction are best examined in clawed frogs, *Xenopus laevis* and *X. tropicalis*. In these amphibian models, EE2-concentrations as low as 0.3 ng/L have been shown to affect calling behavior and mating success^24^. Higher but still environmentally relevant amounts of EE2 (29 to 840 ng/L) have been shown to affect body morphology, metamorphosis and hemoglobin catabolism^25^,^26^. Importantly, EE2 can lead to impaired sexual development as mirrored by gonad histomorphology, demonstrating that male clawed frogs (*X. laevis*) develop mixed sex (=‘intersex’, see below) gonads or even show complete phenotypic sex reversal^26^,^27^,^28^,^29^. The undifferentiated anuran gonad is bipotential and can develop into either ovary or testis^30^. Therefore, exogenous hormones can override the primary genetic sex determination signal and lead to developmental disturbances, mixed sexes or complete sex reversal. One major obstacle of studying EDC-effects in amphibians has been the mostly inaccessible information about genetic sex. In most previous EDC-studies, sex reversal had to be inferred by comparing sex ratios of control and exposed frogs, assuming a normal 1:1 proportion, which may have easily led to wrong conclusions about EDC-impacts on sex ratios. While all amphibian species investigated show genetic sex determination^31^, exhibiting either male (XX/XY) or female (ZZ/ZW) heterogamety, an extrapolated 96% of all species have microscopically indistinguishable sex chromosomes^32^, requiring molecular sexing methods. Although EDC-studies with molecular sexing were applied to the model *Xenopus*^26^,^33^, sex markers have become only recently available for some non-model anurans^32^,^34^,^35^,^36^,^37^,^38^, and have not been used in EDC-experiments.

Using a high-standard flow-through-system and the first direct experimental approach of its kind, we simultaneously exposed European tree frogs (*H. arborea*), green toads (*B. viridis*) and the well investigated but deeply diverged model-species *X. laevis* to EE2, applied molecular sexing followed by histological analysis and compared impacts on their sexual development. We found striking differences in the susceptibility to sex reversal between model and non-model species, showing that state-of-the-art approaches are an important prerequisite to detect species-specific vulnerabilities to EDCs in amphibians.

## Results

### Phenotypic sex reversal of genetic males

**Figure 1:**
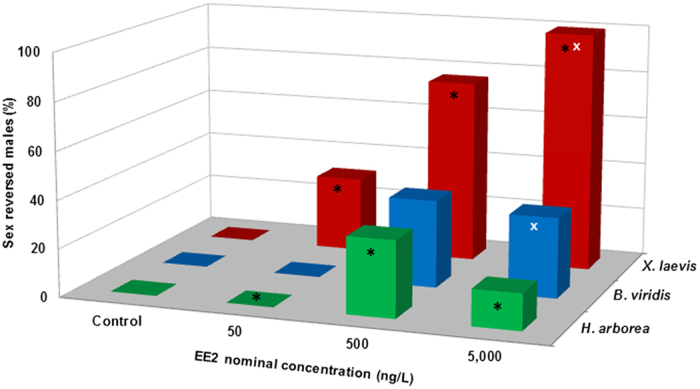
Quantities of sex reversal (contradiction between genetic and phenotypic sex) under the influence of 17α-ethinylestradiol (EE2) in three deeply diverged anuran amphibians. Percentages of genetic-male-to-phenotypic-female sex reversal in African clawed frogs (*Xenopus laevis*, red), European tree frogs (*Hyla arborea*, green), and European green toads (*Bufo viridis*, blue) exposed to three concentrations of EE2 and in control animals; pooled data from two replicate experiments for each treatment or control. Susceptibility differences in genetic-male-to-phenotypic-female sex reversal occurred at all concentrations: (*) significant differences between clawed frogs and tree frogs (p ≤ 0.010); (^x^) significant differences between clawed frogs and green toads (p ≤ 0.001). Statistical analyses were conducted using cross-tabulation, Chi square and Fisher´s exact tests (α = 0.05).

**Figure 2:**
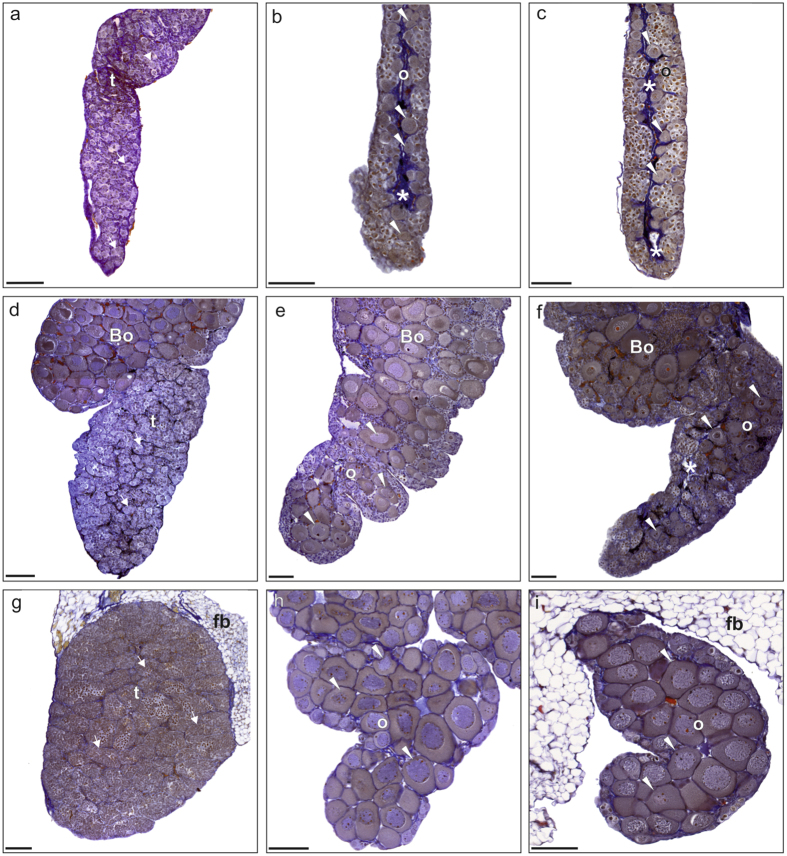
Histological sections of three anuran species under the influence of 17α-ethinylestradiol (EE2). (**a–c**) Normal male, normal female and phenotypically sex-reversed gonad of African clawed frog (*Xenopus laevis*). **(d–f)** Normal male, normal female and phenotypically sex-reversed gonad of European green toad (*Bufo viridis*). **(g–i)** Normal male, normal female and phenotypically sex-reversed gonad of European tree frog (*Hyla arborea*). Bo – Bidder’s organ, characteristic of bufonid gonads (for details: Methods); fb – fat body; o – ovary; t – testis; arrows indicate seminiferous tubules; *ovarian cavity; arrowheads – diplotene oocytes. Scale bars are 100 micrometers.

Among all three anuran species, simultaneous exposure to three EE2-concentrations under flow-through-conditions resulted in different proportions of male-to-female sex reversal, ranging from 15 to 100% (Table 1 and Fig. 1), which was solely revealed when comparing genetic and phenotypic sex of experimental animals. Importantly, no sex reversal occurred in control groups. While sex reversal (Figs 1 and 2) was generally correlated to EE2-concentration, interspecies differences (p ≤ 0.010) between clawed frogs (*X. laevis*) and tree frogs (*H. arborea*) were found at all concentrations, and between clawed frogs and green toads (*B. viridis*) at the highest concentration (5,000 ng/L; p ≤ 0.001). While EE2-treatment produced similar percentages of sex-reversed tree frogs and green toads (15 to 36%), clawed frogs appeared most susceptible (up to 100%). At the lowest concentration (50 ng/L) 31.3% of genetically male clawed frogs developed female phenotypes, i.e. ovaries, while no sex reversal occurred in the non-model species (*H. arborea, B. viridis*). As expected for a feminizing EDC, sex reversal occurred always from genetic male to phenotypic female. According to gross morphological observation and histological evidence, sex-reversed genetic male frogs and toads developed ovaries that showed no difference to those of genetic control and untreated^39^ females.

### Mixed sex gonads

**Figure 3:**
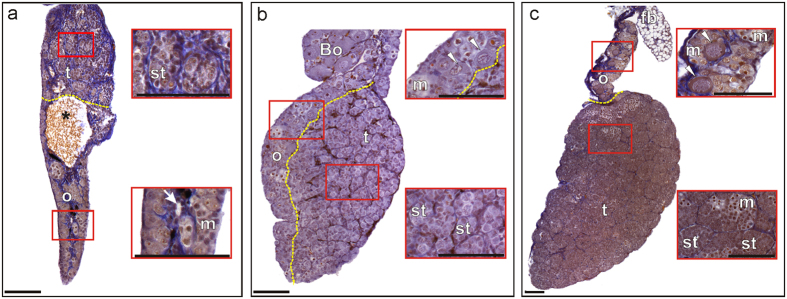
Histological sections of mixed sex gonads of three anuran species under the influence of 17α-ethinylestradiol (EE2). **(a)** African clawed frog (*Xenopus laevis*), **(b)** European green toad (*Bufo viridis*), **(c)** European tree frog (*Hyla arborea*); Fig. 2 for control and sex-reversed individuals. Bo – Bidder’s organ, specific of bufonid toads’ gonads; fb – fat body; m – meiocytes; o – ovary; st – seminiferous tubules; t – testis; *a cavity separating testicular and ovarian parts of the mixed sex gonad; white arrow indicates ovarian cavity in the ovarian portion of the mixed gonad; white arrowheads show diplotene oocytes; yellow dotted lines separate testicular and ovarian parts of the mixed sex gonads. Scale bars represent 100 micrometers.

In addition to sex reversals, EE2-treatment provoked the development of various percentages of mixed sex^40^ gonads (equivalent to ‘intersexes’ of some authors^41^,^42^,^43^) that were histologically recorded in all three species (Fig. 3 and Table 1). Such altered gonads are characterized by the presence of ovarian within testicular tissue in genetic males and were never found in control groups. In contrast to the sex reversal analyses, *X. laevis* formed fewer mixed sex gonads than *B. viridis* (p ≤ 0.026). No significant susceptibility differences between *H. arborea* and the model species were found. Both non-model species also differed in their susceptibility at the lowest concentration (50 ng/L; p ≤ 0.015).

## Discussion

Using a new combination of experimental features, we provide evidence for different quantities of genetic-male-to-phenotypic-female sex reversal in three amphibian species, diverged between 78 million years^44^ (*Hyla, Bufo*) and 206 My (*Xenopus*), under exposure to the estrogen EE2. This synthetic substance is globally of high relevance for EDC-pollution of aquatic ecosystems^14^,^15^. Our new approach combined simultaneous exposure of tadpoles to three EE2-concentrations in a flow-through-system and genetic sexing of metamorphs of model and non-model experimental anurans. We applied environmentally (pollution) and physiologically (expected effects in *X. laevis*) relevant concentrations of EE2. Genetic sexing of metamorphosed tree frogs and green toads revealed these two non-model species to have similar susceptibilities to sex reversal among each other, while both significantly differed from *X. laevis*. This model-species, in which genetic sex is governed by a female heterogametic (ZZ/ZW) chromosome system^45^, proved to be more sensitive to EE2 with a lower dose provoking sex reversals and more affected animals (Table 1). On the other hand, *B. viridis* and *H. arborea*, both diverged 206 My from *X. laevis* and possessing male heterogametic (XX/XY) sex chromosomes^32^,^35^, showed higher percentages of mixed sex individuals than *X. laevis*.

All of this suggests that species-specific developmental stages, sex determination systems or endocrine pathways, shaped by long separate evolutionary histories, were differently affected by EE2, and such a wide spectrum of effects can be generally expected also for other EDCs among diverged anuran lineages.

The occurrence of more than 50% of genetic females among the randomly chosen hatchlings in several of our test tanks underlines the importance of genetic sexing. Unavailability of genetic sexing, as in many previous studies, could easily lead to wrong conclusions about the strength of feminizing (or masculinizing) effects of EDCs when determining “no observed effect concentration” (NOEC) and “lowest observed effect concentration” (LOEC) for endocrine active substances.

Different estrogenic compounds with concentrations reaching from the low nanogram- to the high microgram-per-liter range have been shown to provoke phenotypic male-to-female sex reversals in *X. laevis*^46^,^47^,^48^ models. To our knowledge, only one previous study^26^ has examined sex reversals after EE2-exposure using molecular sexing in *X. laevis*, examining a similar range of concentrations (90, 840, 8,810 ng/L). In contrast to our study, male-to-female sex reversals were not detected under the 90 ng/L treatment, and at the higher concentrations with only 7 and 17%, respectively. However, these authors used a static and not a flow-through-system, which may explain the deviating results to our study, as EE2-concentrations may stronger fluctuate due to effects of metabolic activity of microorganisms in tanks^49^,^50^, due to greater biomass sorption of EE2^51^, or due to simple adsorption to surfaces of exposure tanks. Beyond the synthetic EE2, on which we focused due to its high environmental relevance, previous sex reversal estimates in *X. laevis*, evaluating only sex ratios, involved the natural, ephemeral 17β-estradiol (E2). Such E2-treatments provoked skewed sex ratios^40^,^48^,^52^,^53^,^54^ or complete feminization^46^,^53^,^55^,^56^. In *H. arborea* and *B. viridis* effects have only been studied^56^ at the very high 100,000 ng/L E2-concentration. In both species, no female-biased sex ratios but a high percentage (59.3%) of undifferentiated gonads in *B. viridis* were found. Since gonad differentiation in bufonid toads is slower compared to the other species at this developmental stage^39^, we assume that the time of dissection at metamorphosis may have influenced these results.

Several inconsistent outcomes in the literature may be explicable by the potentially wrong assumption of initial 1:1 sex ratios of experimental amphibians. Based on our data, we strongly recommend genetic sexing, whenever available, as a hallmark of appropriate evaluation of EDC-effects in amphibians. This demand can be extended to other vertebrates and generalized to EDC-research in organisms with homomorphic sex chromosomes, including invertebrates^57^. Otherwise, as shown here, complete sex reversal as a very profound EDC-effect, occurring at low concentrations, may be completely overlooked. Furthermore, deep phylogenetic differences may result in strong susceptibility differences towards EDCs. Though we do not advocate the extensive use of endangered amphibians, we conclude that results gained from earlier studies in *X. laevis* in general and without genetic sex information specifically should not be uncritically extrapolated to other anuran species.

## Methods

### Animals

This experiment was approved by the German State Office of Health and Social Affairs (LaGeSo, Berlin, Germany; G0359/12); all methods were carried out in accordance with approved guidelines. *Xenopus laevis* tadpoles were obtained from the stock at the Leibniz-Institute for Freshwater Ecology and Inland Fisheries. Induction of spawning and tadpole husbandry followed standard methods^58^. Parental animals of *B. viridis* and *H. arborea* were caught at several localities in Greece (Supplementary Table 1), and non-invasively DNA-sampled^59^. Parts of their clutches were transferred to IGB (permit 115790/229) and acclimated at 22 ± 1 °C in 10 L Milli-Q grade water, supplemented with 2.5 g marine salt (Tagis, Germany).

### Hormone exposure and experimental conditions

17α-Ethinylestradiol (Sigma-Aldrich, Germany), dissolved in dimethyl sulfoxide (DMSO; Roth, Germany), was applied in nominal concentrations of 50, 500 and 5,000 ng/L (Supplementary Fig. 1 and Supplementary Table 2, for measurements during the experiment); control animals received 0.00001% DMSO. EE2-concentrations in test tanks were checked weekly by high performance liquid chromatography/mass spectrometry (HPLC-MS/MS), and adjusted if required. In order to minimize adsorption or release of EDCs, we used glass tanks and all connections of the flow through system consisted of inert materials involving mainly PTFE (Polytetrafluoroethylene, “Teflon”)-coating or Platinum-cured Silicon tubing (Cole-Parmer). Exposure of tadpoles started at Gosner^60^-stage 22–23 in *B. viridis* and *H. arborea*, equivalent to Nieuwkoop-Faber^61^ stage 42–44 in *X. laevis*, distinctly prior to the sensitive phase of sex determination in all species^30^,^62^. Twenty randomly chosen individuals per species and treatment were transferred into each test tank in a high-standard flow-through-system (details^52^). Two replicates per exposure group (including control) comprised in total 160 tadpoles per species. Stock solutions and water were piped via a peristaltic pump into a mixing chamber, mixed to final EE2-concentrations, and supplied to a cluster of three test tanks each. Concentrations were thus identical for all three species in each treatment group. Tadpoles were reared in a 12/12 h light/dark cycle at constantly 22 ± 1 °C in sufficiently aerated and regularly cleaned tanks. Weekly monitored water parameters comprised: dissolved oxygen, nitrate, ammonium, pH, conductivity, and hardness; values were adequate as in previous studies involving the same equipment^40^. Tadpoles were fed SeraMicron (Sera, Germany), *H. arborea* and *B. viridis* were additionally supplied with TetraMin (Tetra, Germany). To imitate natural conditions under which *H. arborea* and *B. viridis* leave water at metamorphosis, animals were transferred to glass terraria at Gosner stage 46. *Xenopus laevis* were dissected at equivalent Nieuwkoop-Faber stage 66; hylids and bufonids after sufficient post-metamorphic differentiation^39^.

### Phenotypic sexing based on gonad gross morphology and histology

Animals were anesthetized by immersion in tricaine methanesulfonate (MS 222; Sigma-Aldrich), decapitated and dissected under a binocular microscope (Olympus SZX7). Gonadal anatomy served for preliminary phenotypic sexing and detection of underdeveloped gonads. To improve visualization, a drop of Bouin’s solution (Sigma-Aldrich) was added; and *in situ* anatomical photographs were taken (Olympus DT5 camera). For histology, gonads were carefully dissected, separated from adjacent tissue, fixed in Bouin (24 h) and subsequently rinsed several rounds in 70% ethanol. Histological sections were prepared for 50% of study animals (from each tank 10 randomly chosen individuals, i.e. 20 per treatment group). Analyses were performed according to established protocols^30^,^39^,^63^. Using Stemi SV11 (Zeiss) microscope and camera, separated gonads were photographed, embedded in paraplast, sectioned into 7 μm longitudinal slices, stained with Mallory’s trichrome, and examined using Zeiss Axioskop 20 microscope. Images were acquired by a cooled Carl Zeiss AxioCam HRc CCD camera. Histological sections were screened slide by slide to establish phenotypic sex. Ovaries were recognized by the presence of ovarian cavities, early meiotic oocytes and/or diplotenes, and testes by spermatogonia, spermatocytes and/or seminiferous cords or tubules. In the case of *B. viridis*, the most anterior part of both male and female gonads is Bidder’s organ, an ovary-like structure, characteristic of bufonids^64^. In *B. viridis* mixed sex was defined when ovarian meiocytes were found inside male testicular tissue behind the physiological transition region between Bidder’s organ and the actual gonad. All phenotypic sexing was performed without prior knowledge about genetic sex of animals.

### Genotypic sex determination

DNA extraction involved the BioSprint robotic workstation with its 96 DNA Plant Kit (Qiagen, Germany) according to the manufacturer’s protocol. To establish genetic sex, species-specific polymerase chain reactions (PCRs) were conducted on Eppendorf Mastercyclers (Ep Gradient S). For *X. laevis*, two genes were amplified^45^,^65^: *DMRT1* and the female-specific *DM-W* (Supplementary Table 3). Genetic sexing of non-model species involved microsatellites WHa5–201 and Ha-H108^34^,^36^ (*H. arborea*) and C201^32^,^66^, HNRNPD and CHD1^67^ (*B. viridis*; Supplementary Table 3). Genotypes were analyzed on a sequencer (3500 × L Genetic Analyzer, Applied Biosystems) and Genemapper v. 4.0 was used for visualization of peaks. DNA quality issues (four *H. arborea*) and homomorphy of microsatellites (33 *B. viridis*) prevented genetic sexing in these individuals that were excluded from sex reversal analyses.

### Detection of complete sex reversal and mixed sex

Phenotypic sexing of all animals was based on gross morphology and histology of gonads^39^,^52^. Complete sex reversal was stated if genetic males showed a phenotypically female gonad, irrespective of the degree of its differentiation and not differing from those of control females. Mixed sex gonads were detected by the presence of ovarian and testicular tissue in the same gonad.

### Statistics

All data were analyzed with SPSS Statistics 22 (IBM, Armonk, NY). Intra- and inter-specific differences in EE2-susceptibility were examined. For evaluations of sex reversal and mixed sex, we first compared both replicates per species and parameter using Fisher’s exact test. If no differences (exact p ≥ 0.05) were found, both replicates per treatment were pooled in order to compare control and exposure groups within and between species using cross-tabulations with 2-sided Chi square tests (α = 0.05). Post-hoc Fisher’s exact tests (2-sided) were applied for pairwise comparisons including False Discovery Rate corrections^68^.

## Additional Information

**How to cite this article**: Tamschick, S. *et al*. Sex reversal assessments reveal different vulnerability to endocrine disruption between deeply diverged anuran lineages. *Sci. Rep.* **6**, 23825; doi: 10.1038/srep23825 (2016).

## Supplementary Material

Supplementary Information

## Figures and Tables

**Table 1:** Effects of three 17α-ethinylestradiol (EE2) concentrations (50, 500 and 5,000 ng/L) on the sexual development of model and non-model amphibian species.

*X. laevis*	Genet. sexed	Females	Males	Females	Males	Sex-reversed		Mixed sex	
	N	N	N	%	%	N	%	N	%
Control	35	24	11	68.6	31.4	0	0	0	0
50 ng/L	38	22	16	57.9	42.1	5*	31.3*	0^x^	0.0^x^
500 ng/L	37	20	17	54.1	45.9	13*,#	76.5*,#	1	12.5
5,000 ng/L	38	21	17	55.3	44.7	17*,^x^, #	100*,^x^, #	0^x^	0.0^x^
***H. arborea***									
Control	36	13	23	36.1	63.9	0	0	0	0
50 ng/L	36	15	21	41.7	58.3	0*	0.0*	0°	0.0°
500 ng/L	41	22	19	53.7	46.3	6*,#	31.6*,#	3	30
5,000 ng/L	37	17	20	45.9	54.1	3*	15.0*	3	27.3
***B. viridis***									
Control	25	13	12	52	48	0	0	0	0
50 ng/L	24	11	13	45.8	54.2	0	0	4^x^,°	57.1^x^,°
500 ng/L	27	15	12	55.6	44.4	4	36.4	4#	80.0#
5,000 ng/L	27	12	15	44.4	55.6	5^x^	33.3^x^	9^x^,#	69.2^x^,#

Species comprised African clawed frogs (*Xenopus laevis*), European tree frogs (*Hyla arborea*), and European green toads (*Bufo viridis*); numbers and percentages of genetically sexed individuals, sex-reversed males and mixed sex individuals. Significant inter-species susceptibility differences occurred at all concentrations, resulting in genetic-male-to-phenotypic-female sex reversal and development of mixed sex individuals.

^*^Significant difference between clawed frogs and tree frogs;

^x^between clawed frogs and green toads;

^°^between tree frogs and green toads;

^#^significant difference between treatment and control groups within the same species.
