# Supplementary material for: Sex reversal assessments reveal different vulnerability to endocrine disruption between deeply diverged anuran lineages
Source: Sci Rep. 2016 Mar 31;6:23825. doi: 10.1038/srep23825 (PMC4814869; doi:10.1038/srep23825)

Supplementary Information

<u>Title:</u>

Sex reversal assessments reveal different vulnerability to endocrine disruption between deeply diverged anuran lineages

<u>Authors:</u>

Stephanie Tamschick, Beata Rozenblut-Kościsty, Maria Ogielska, Andreas Lehmann, Petros Lymberakis, Frauke Hoffmann, Ilka Lutz, Werner Kloas, Matthias Stöck

Supplementary Table 1: **Geographic coordinates for origin of European tree frogs (*Hyla arborea*) and European green toads (*Bufo viridis*).**

<table>
<tr><th>Family ID</th><th>Species</th><th>Locality</th><th>Long.</th><th>Lat.</th><th>Number of individuals</th></tr>
<tr><td rowspan="2">Ha_Mix</td><td rowspan="2">H. arborea</td><td>Greece, Crete, Thrapsano</td><td>35.185</td><td>25.303</td><td rowspan="2">40 siblings and 40 mixed offspring</td></tr>
<tr><td>Greece, Crete, Mochos</td><td>35.247</td><td>25.442</td></tr>
<tr><td rowspan="2">Ha_Mo×La</td><td rowspan="2">H. arborea</td><td>Greece, Crete Mochos (mother)</td><td>35.247</td><td>25.442</td><td rowspan="2">Mother, father, 80 siblings</td></tr>
<tr><td>Greece, Crete Lasithi Plateau (father)</td><td>35.193</td><td>25.432</td></tr>
<tr><td rowspan="2">Bv_Mix</td><td rowspan="2">B. viridis</td><td>Greece, Crete, Thrapsano</td><td>35.185</td><td>25.303</td><td rowspan="2">40 and 32 siblings</td></tr>
<tr><td>Greece, Naxos</td><td>36.975</td><td>25.493</td></tr>
<tr><td>Bv_GR13_11x12</td><td>B. viridis</td><td>Greece, Crete, Lasithi Plateau</td><td>35.193</td><td>25.432</td><td>Mother, father, 80 siblings</td></tr>
</table>

Supplementary Table 2: Measured ethinylestradiol (EE2) concentrations. In weeks 4, 5 and 8 only stock solutions were quantified; chart in Supplementary Fig. 1.

Concentration EE2 ng/L	Week 1	Week 2	Week 3	Week 6	Week 7	Week 9	Week 10	Average
5,000	2950	3030	4030	5000	4910	4680	3850	4064
500	670	1270	1275	260	480	357		719
50	80	110	40	100	30	30	20	59

Supplementary Table 3: **Primer information and PCR protocols for molecular sexing of *Xenopus laevis*, *Hyla arborea* and *Bufo viridis*. (a)** Marker with corresponding accession numbers, sequences, labels and sizes for all three species. **(b)**, **(c)** PCR ingredients and cycler protocol for genes *DMRT1* and *DM-W* for *X. laevis*. **(d)**, **(e)** PCR ingredients and cycler protocol for microsatellite markers WHa5-201 and Ha-H108 for *H. arborea*. **(f)**, **(g)** PCR ingredients and cycler protocol for microsatellite marker C201 for *B. viridis*. **(h)**, **(i)** PCR ingredients and cycler protocol for microsatellite markers HNRNPD and CHD1 for *B. viridis*.

(a)

**Species**	**Locus**	**GenBank Acc. number**	**Primer sequences (5'=>3')**	**Fluores-cence label**	**Allele size (bp)**
X. laevis	*DMRT1*	AB259777	F: AACAGGAGCCCAATTCTGAG	none	206
			R: AACTGCTTGACCTCTAATGC		
X. laevis	*DM-W*	AB365520	F: CCACACCCAGCTCATGTAAAG	none	260
			R: GGGCAGAGTCACATATACTG		
B. viridis	C201	HQ386137	F: AGGACCCAGGATTTCCAT	HEX (green)	94 - 122
			R: GCTTCTACCAAAGACTGTTCC		
B. viridis	HNRNPD	SAMN03993917 (Bioproject)	F: AGCGTGATGGAGGCGGCCGCCAGCG	FAM (blue)	128 - 140
			R: CTTGCTGGCGTTGATCTTC		
B. viridis	CHD1	SAMN03993917 (Bioproject)	F: TCCCTGCATTCTGTCAAGAAC	HEX (green)	299 - 301
			R: TGGACGGTATGAGGAACCAT		
H. arborea	Ha-H108	EU029102	F: GGGGGTGAGTAAGGGTTAAATC	FAM (blue)	234 - 261
			R: GCCACTGTATAGTCCCTCCCTA		
H. arborea	WHa5-201	AJ403999	F: TCATGGACTGTCGTCATGGT	HEX (green)	228 - 253
			R: AGGTAAATGGAATCTGGGTGTG		

(b)

	**Volume per well in µl**
ddH_2O	19.25
10x Buffer (15 mM $MgCl_2$)	2.5
dNTP Mix (2,5 mM)	0.25
Taq polymerase	0.2
Primer *DM-W* for	0.2
Primer *DM-W* rev	0.2
Primer *DMRT1* for	0.2
Primer *DMRT1* rev	0.2
**Total Volume**	**23.00**
**DNA**	**2.00**

(c)

**Cycle**	**Temperature**	**Time period**	**Process**
1	95°C	4 min	Denaturation
2	95°C	1 min	Denaturation
3	60°C	1 min	Annealing
4	72°C	1 min	Extension
5	go to 2	30 x	
6	72°C	10 min	Extension
7	10°C	forever	

(d)

	**Volume per well in µl**
ddH_2O	3.18
Multiplex Mastermix	3.00
Primer Ha5-201 for	0.33
Primer Ha5-201 rev	0.33
Primer H108 for	0.08
Primer H108 rev	0.08
**Total Volume**	**7.00**
**DNA**	**3.00**

(e)

**Cycle**	**Temperature**	**Time period**	**Process**
1	95°C	15 min	Denaturation
2	94°C	30 s	Denaturation
3	58°C	1.5 min	Annealing
4	72°C	1.0 min	Extension
5	go to 2	32 x	
6	60°C	30 min	Extension
7	10°C	For ever	

(f)

	**Volume per well in µl**
ddH_2O	1.86
Multiplex Mastermix	3.00
Primer D214 for	0.07
Primer D214 rev	0.07
Primer C201 for	1.50
Primer C201 rev	1.50
**Total Volume**	**8.00**
**DNA**	**2.00**

(g)

**Cycle**	**Temperature**	**Time period**	**Process**
1	95°C	15 min	Denaturation
2	94°C	30 s	Denaturation
3	48°C	1.5 min	Annealing
4	72°C	1.0 min	Extension
	go to 2	35 x	
5	60°C	30 min	Extension
6	10°C	For ever	

(h)

	**Volume per well in µl**
ddH_2O	2.90
10x Buffer (15 mM $MgCl_2$)	1.00
Sol Q	1.00
dNTP Mix (2,5 mM)	1.00
Top Taq DNA polymerase	0.10
Primer HNRNPD for	0.50
Primer HNRNPD rev	0.50
Primer CHD1 for	0.50
Primer CHD1 rev	0.50
**Total Volume**	**9.00**
**DNA**	**1.00**

(i)

**Cycle**	**Temperature**	**Time period**	**Process**
1	95°C	3 min	Denaturation
2	94°C	30 sec	Denaturation
3	59,5°C	60 sec	Annealing
4	72°C	40 sec	Extension
	go to 2	35 x	
5	72°C	10 min	Extension
6	10°C	For ever	

Supplementary Fig. 1: Measured 17α-ethinylestradiol (EE2) concentrations. Nominal EE2 concentrations comprised 50 (green line), 500 (red line) and 5,000 (blue line) ng/L; for numeric measured values: Supplementary Table 2.

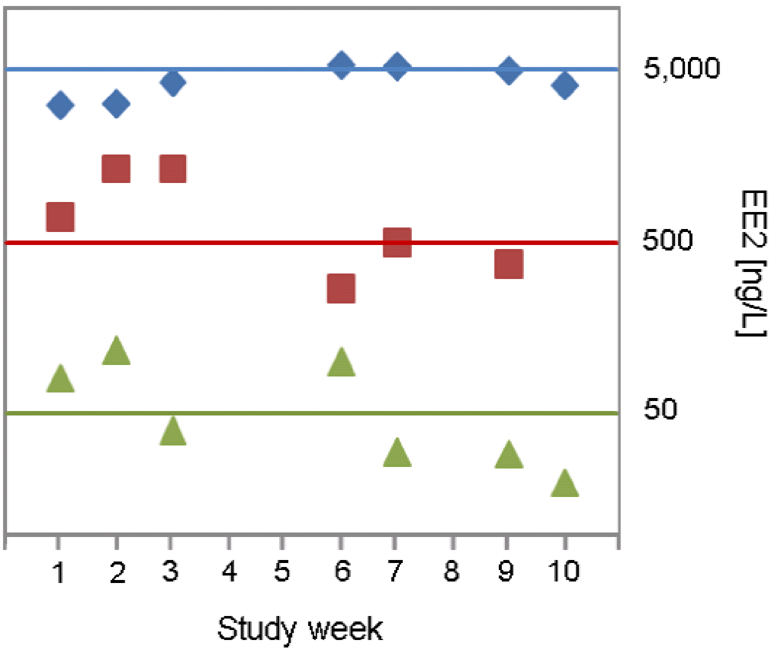

Supplement: Supplementary Information [file srep23825-s1.pdf]
